# A Rare Cutaneous Tumor With Unusual Dermoscopic Features

**DOI:** 10.1002/ccr3.70598

**Published:** 2025-07-30

**Authors:** Antonio Di Guardo, Luca Gargano, Andrea Ascione, Domenico Giordano, Flavia Persechino, Severino Persechino

**Affiliations:** ^1^ Unit of Dermatology Sant'Andrea Hospital, Sapienza University of Rome Rome Italy; ^2^ IDI‐IRCCS Dermatological Research Hospital Rome Italy; ^3^ Department of Experimental Medicine Sapienza University of Rome Rome Italy; ^4^ Unit of Dermatology Policlinico Umberto I, Sapienza University of Rome Rome Italy

**Keywords:** adnexal neoplasms, dermopathology, dermoscopy, mixed tumor of the skin, skin cancer

## Abstract

Although subtle, dermoscopic clues such as milia‐like cysts, white structureless areas, and peripheral vessels may suggest a biphasic adnexal tumor. Histopathology remains essential for definitive diagnosis. Complete excision with clear margins is typically curative. Rare tumors like CS may benefit from AI‐driven tools to improve preoperative recognition.

## 1 Introduction

Chondroid syringoma (CS) is a rare, mixed tumor of sweat gland origin, typically presenting as a firm, painless nodule. It requires a combination of clinical examination, dermoscopy, and histopathology for accurate diagnosis [1, 2]. Surgical excision is the main treatment, with histopathological evaluation essential for distinguishing benign from malignant forms.

## 2 Case History

**FIGURE 1:**
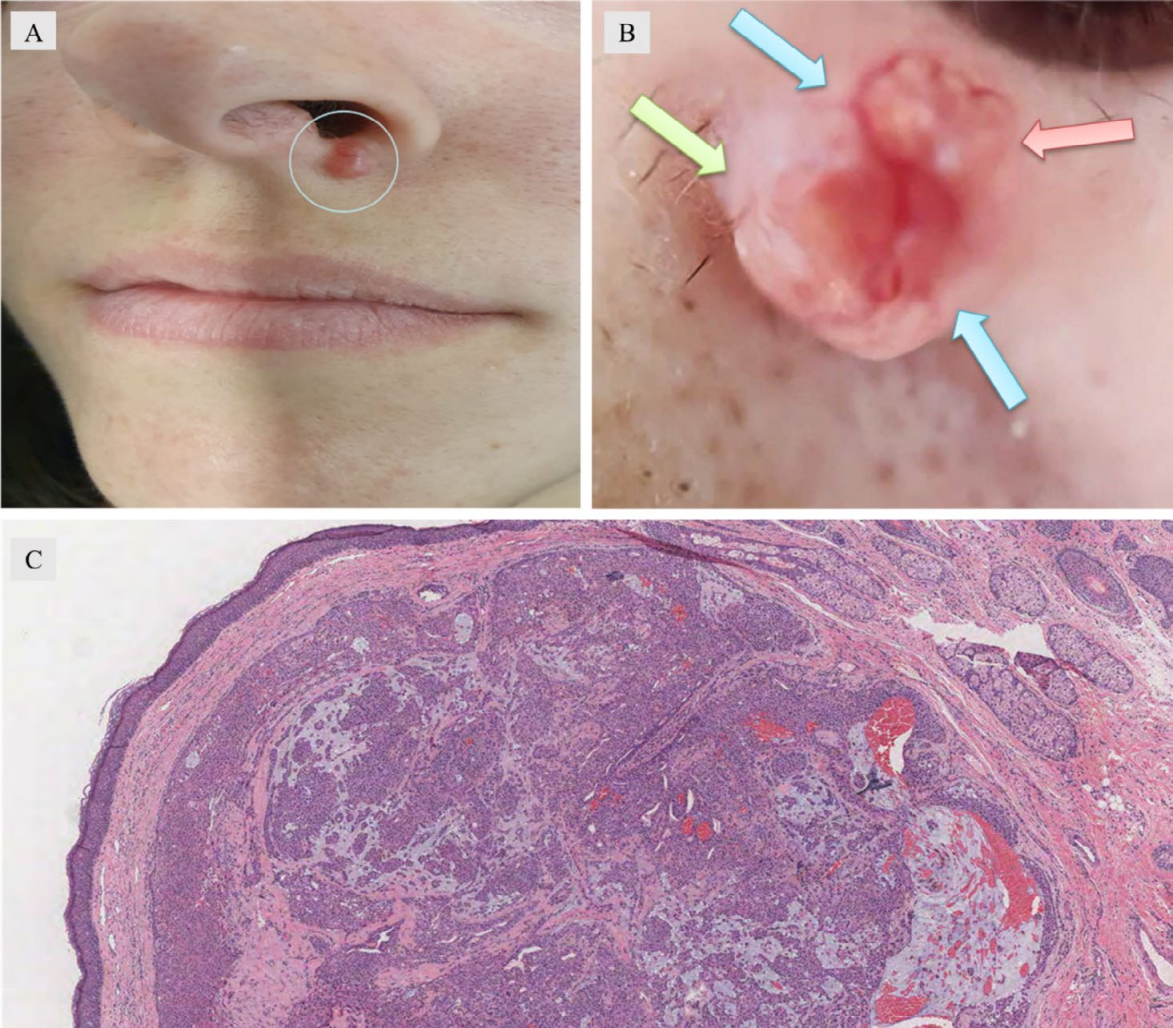
(A) Solitary firm, erythematous nodule on the upper left side of the philtrum. The surface of the nodule showed multiple white‐yellowish areas and telangiectasia. (B) Dermoscopy under polarized mode showing white structureless area (green arrow), milia‐like cysts (red arrow), and a “ring” of vessels (blue arrows). (C) Dermal unencapsulated lesion with lobular proliferation of tumor cells with glandular differentiation (hematoxylin and eosin, 20×).

A 28‐year‐old woman presented with an asymptomatic, slowly enlarging lump on her left nasolabial fold over the past 2 years. Her medical history was unremarkable, and no prior trauma to the area was reported. On examination, a solitary, dome‐shaped, firm nodule measuring 12 × 10 mm was noted. The nodule's surface was irregular, with prominent telangiectasias and white‐yellowish areas. There was no regional lymphadenopathy or systemic involvement (Figure 1A). Polarized dermoscopy revealed distinctive findings, including prominent telangiectatic vessels forming a ring around the lesion, milia‐like cysts, and “cotton‐white” areas (Figure 1B). Surgical excision under local anesthesia was performed. Histopathological examination (Figure 1C) revealed an unencapsulated dermal lesion with multilobulated contours. High magnification showed biphasic features: epithelial and myoepithelial cells arranged in branching tubular structures and solid sheets, embedded within a chondromyxoid stroma. Glandular differentiation was evident in the epithelial component.

## 3 Differential Diagnosis

Several skin neoplasms can present with overlapping clinical and dermoscopic features, making the diagnosis of CS particularly challenging. In this case, the clinical features may suggest other adnexal or epithelial tumors. The dermoscopic aspects added further complexity to the differential. Basal cell carcinoma (BCC) was initially considered due to the presence of telangiectasias and a nodular architecture. However, BCC typically shows central ulceration, rolled borders, and arborizing vessels on dermoscopy, which were not observed in this case [3]. Pilomatricoma can also present as a firm, slowly growing nodule with dermoscopic milia‐like cysts and hairpin vessels. Nonetheless, the absence of the characteristic bluish hue and the presence of a chondromyxoid stroma on histology ruled it out [4]. Sebaceoma may display cystic structures and telangiectasias but usually lacks the glandular and myoepithelial architecture with chondromyxoid matrix seen in this case [5]. Its sebaceous differentiation on histology allows a clear distinction. This combination of clinical, dermoscopic, and histopathological findings—especially the ring‐like telangiectasias, cotton‐white areas, and biphasic epithelial/myoepithelial proliferation in a chondromyxoid stroma—is strongly suggestive of CS and rare in facial locations in young adults.

## 4 Results

After conducting a thorough clinical examination, dermoscopy, and histopathological analysis, the lesion was diagnosed as CS. Histopathology revealed a biphasic tumor with both glandular and cartilaginous components, which confirmed the diagnosis. The lesion was unencapsulated, with a dermal location and multilobulated contours, which is typical for CS. Postsurgical follow‐up showed no evidence of recurrence after the excision of the lesion. Given the benign nature of most CS cases, routine follow‐up was recommended for the first year, including clinical examination every 3–6 months. No signs of metastasis or recurrence were observed at the 6‐month follow‐up visit. Given the rarity of CS and its potential for malignancy in some cases, continued monitoring is advised for any signs of recurrence, especially in the event of incomplete excision. Ultrasound of the regional lymph nodes and radiography of the chest and abdomen showed no evidence of metastasis.

## 5 Discussion

CS is a rare adnexal neoplasm of mixed epithelial and mesenchymal origin, representing less than 0.2% of all skin tumors [1, 2]. While typically benign, a malignant variant (MCS) has been described, more frequently affecting the extremities and associated with local recurrence and distant metastasis in up to 19% of cases [6]. In contrast, benign CS tends to present as a slow‐growing, firm nodule in the head and neck region, as observed in our young patient with an unusual localization on the nasolabial fold. The clinical and dermoscopic features in this case—ring‐like telangiectasias, milia‐like cysts, and “cotton‐white” areas—reflect the tumor's biphasic architecture with epithelial and myoepithelial components embedded in a chondromyxoid matrix. These findings, although nonpathognomonic, may contribute to future efforts to delineate dermoscopic patterns specific to CS.

In terms of diagnostic work‐up, no radiologic imaging was deemed necessary in our case, given the lesion's superficial location, small size, and absence of clinical signs suggestive of deep invasion. However, in cases of larger, deeper, or rapidly growing lesions, imaging studies may be valuable to better characterize the extent of the tumor and assist in surgical planning [7]. High‐resolution ultrasound is typically the first‐line modality, offering a noninvasive assessment of soft tissue involvement. In more complex cases, particularly when there is suspicion of malignancy or when the tumor is located in anatomically critical areas, CT or MRI can provide additional information regarding local infiltration or involvement of adjacent structures. It should be noted that, given the rarity of CS, there is limited data regarding the role of fine‐needle aspiration biopsies (FNAB) in its preoperative diagnosis. Available evidence suggests that FNAB may frequently lead to incorrect or inconclusive results. Definitive diagnosis and malignancy exclusion typically rely on histopathologic examination, which reveals nests of epithelial and myoepithelial cells embedded in a characteristic chondromyxoid stroma.

Although CS remains a rare and often unexpected histological diagnosis, future advances in artificial intelligence and machine learning may offer new opportunities for earlier and more accurate identification. Recent developments in deep learning–based systems, such as SNC_Net, have demonstrated remarkable accuracy in classifying dermoscopic images of various skin cancers by integrating handcrafted and convolutional features [8]. Similarly, multistage and multiclass convolutional neural network frameworks have been shown to improve the subclassification of benign and malignant skin lesions, with potential applicability even to rare tumors like CS, particularly in distinguishing them from clinically similar conditions [9]. Furthermore, optimization algorithms, when coupled with neural networks, may further enhance diagnostic precision by supporting clinical decision‐making even in atypical or complex cases [10]. While these technologies are not yet validated for CS specifically, their integration into dermatologic workflows could, in the future, aid in recognizing unusual dermoscopic features suggestive of adnexal tumors and improve preoperative diagnostic accuracy in challenging cases.

From a therapeutic standpoint, complete surgical excision with histologically negative margins remains the standard treatment for benign CS. Although there is no universally established surgical margin, excision with 3–5 mm of clinically normal‐appearing skin is generally considered adequate for well‐circumscribed benign lesions, as also recommended for nonmelanotic skin cancers of similar size [11]. In our case, excision was performed with 4 mm margins, and histological examination confirmed complete tumor removal. The use of Mohs micrographic surgery (MMS) for CS is still a matter of debate. MMS offers superior margin control and maximal tissue preservation, which can be particularly beneficial in cosmetically sensitive areas or when lesions have ill‐defined borders. Moreover, although rare, malignant variants of CS have been reported, and their distinction from benign forms may not always be possible based solely on clinical or imaging findings preoperatively [12]. In such situations, MMS could theoretically offer an additional safeguard by ensuring complete excision of any microscopically infiltrative or satellite tumor cells, which are features more typical of malignant lesions. However, in cases like ours—characterized by clear clinical demarcation and confirmed benign histology—conventional excision with negative margins is considered sufficient, and the routine use of MMS is not currently justified. As for radiotherapy (RT), current evidence does not support its use as primary treatment for CS. In the few reported cases where RT was applied, it was generally administered after the development of metastases rather than as part of the initial therapeutic strategy [12]. Its efficacy in this context remains uncertain, and no significant benefit has been demonstrated in preventing recurrence when used concomitantly with surgery.

As for follow‐up, given the benign nature of the lesion and the histologically confirmed complete excision with negative margins, a conservative clinical approach was adopted. However, it is important to note that there is currently no standardized or universally accepted follow‐up protocol for CS in the literature. In benign cases like ours, we suggest periodic clinical evaluation every 6–12 months for at least 2 years, especially in younger patients or when there are concerns such as histologic atypia, close margins, or incomplete excision. In contrast, malignant CS, although rare, are associated with a high rate of local recurrence and distant metastasis. In such cases, a more intensive follow‐up regimen is warranted. This may include imaging studies such as regional lymph node ultrasound or PET/CT scans to monitor for subclinical disease progression, particularly in the early postoperative period. The follow‐up strategy should therefore be tailored to the individual risk profile, taking into account both histologic features and clinical behavior.

In conclusion, this case underscores the diagnostic complexity of CS and highlights the potential value of dermoscopy in raising suspicion for adnexal neoplasms. It also reinforces the importance of a tailored, multidisciplinary approach that balances oncologic safety with aesthetic and functional considerations. Given the rarity of CS and the limited dermoscopic literature, this case contributes novel features that may help expand the diagnostic spectrum of this uncommon tumor. Future studies should aim to clarify optimal surgical margins, indications for Mohs surgery, and standardized follow‐up protocols.

## Multiple Choice Questions

1. What is the most common dermoscopic feature of chondroid syringoma?
  - Pigmented blotches
  - Whitish areas and telangiectasias
  - Arborizing vessels
  - Yellowish structureless areas
**Answer:** b. Whitish areas and telangiectasias
2. Which treatment is most appropriate for a suspected malignant chondroid syringoma?
  - Standard excision with narrow margins
  - Cryotherapy
  - Wide local excision
  - Topical chemotherapy
**Answer:** c. Wide local excision

## Author Contributions

**Antonio Di Guardo:** writing – original draft. **Luca Gargano:** writing – original draft. **Andrea Ascione:** writing – original draft. **Domenico Giordano:** writing – original draft. **Flavia Persechino:** writing – original draft. **Severino Persechino:** writing – original draft.

## Consent

Written informed consent has been obtained from the patient to publish this paper.

## Conflicts of Interest

The authors declare no conflicts of interest.

## Data Availability

All data reported in the present manuscript will be available on request from the authors.
